# Supplementary material for: Are carbon emissions trading and green financial instruments synergistic? -Comprehensive quantitative research based on content analysis
Source: PLoS One. 2024 Mar 7;19(3):e0298601. doi: 10.1371/journal.pone.0298601 (PMC10919601; doi:10.1371/journal.pone.0298601)
Supplement: S1 Data — (ZIP) [file pone.0298601.s001.zip › Data/地级市Z_F工作报告.zip!/╕╩╦α/└╝╓▌╩╨/└┤╫╘╣1⁄2╓┌║┼í╛┬φ┐╦╩2╛▌═°í┐_2017.docx]

各位代表：

　　现在，我代表市人民政府向大会作工作报告，请予审议，并请各位政协委员和其他列席会议的同志提出意见。

　　一、过去五年政府工作回顾

　　五年来，按照省委、省政府建设幸福美好新甘肃的目标和“做大做强做美兰州”的要求，在市委的坚强领导下，全市上下聚焦中心带动，奋力攻坚克难，加速推动发展，完成了“十二五”规划和本届政府的各项目标任务，开创了经济社会发展的崭新局面，奠定了全面建成小康社会的坚实基础。奋进中的兰州，展现出一幅发展提速、产业升级、民生改善、社会和谐的美好画卷。

　　——这五年是“做大兰州”卓有成效的五年，中心带动的空间格局不断拓展。编制实施第四版城市总体规划，中心城区规划面积由221平方公里扩大到741平方公里。抢抓“国办47条”政策机遇，获批建设国家级兰州新区，形成老城与新区互动发展的“大兰州”格局。高新区、经济区实现增容扩区，东部科技城、青白石等重点区域开发积极推进。建成西客站、兰新高铁和中川城际铁路，铁路交通跨入高铁时代，中川机场升级为国际机场，国际和地区航线达到21条，兰州融入区域发展大格局的步伐明显加快。

　　——这五年是“做强兰州”卓有成效的五年，中心带动的实力水平大幅提升。全市生产总值年均增长10.73%，预计今年达到2220亿元以上，是2011年的1.6倍，占全省的比重由27.09%提升到30%以上。固定资产投资、社会消费品零售总额、第三产业增加值、一般公共预算收入、农村居民人均可支配收入实现翻一番，城镇居民人均可支配收入提高85%。全市300个贫困村实现整体脱贫，累计减贫29万人，贫困发生率由24%下降到1%以下。五年招商引资累计到位资金7300多亿元，引进各类500强企业56家，为做强兰州带来了新的支撑。

　　——这五年是“做美兰州”卓有成效的五年，中心带动的环境形象明显改善。特别是面对长期严重的大气污染，在国家的大力支持下，省市联动、全民行动，齐心协力向大气污染宣战，打造了国内外瞩目的“兰州蓝”，创造的“兰州经验”得到国家充分肯定，在巴黎世界气候大会上荣获“今日变革进步奖”，被“中国经济生活大调查”评为2015年度十大幸福城市之一。成功举办兰州国际马拉松赛、中国金鸡百花电影节、敦煌行·丝绸之路国际旅游节等大型节会赛事，兰州国际马拉松赛成为国内马拉松十大金牌赛事和国际田联铜牌赛事，向国内外充分展示了“黄河之都、金城兰州”的美好形象。今年接待国内外游客可达5150万人次，实现旅游总收入435亿元，分别是2011年的3.66倍和4.23倍。中川机场旅客吞吐量连年大幅增长，截至今年11月28日突破1000万人次，是2011年的3倍。

　　五年来，全市上下牢牢扭住发展第一要务，加快转变经济发展方式，产业结构实现突破性转变。预计三次产业结构由2011年的2.94∶48.27∶48.79调整为2.7∶34.82∶62.48，第三产业比重提高13.69个百分点。非公经济增加值年均增长22.7%，预计达到996亿元，占生产总值的比重由38.3%提高到44.9%。战略性新兴产业加快成长，预计增加值达到296亿元，占生产总值的比重为13.3%。文化产业增加值达到70亿元，是2011年的2.82倍。电子商务蓬勃兴起。大数据产业发展势头良好。

　　循环经济加快发展，全国节能减排财政政策综合示范城市、国家级“城市矿产”示范基地等创建工作深入推进。金融助推经济发展的作用日渐增强，全市金融机构人民币各项存款余额、贷款余额可达8742亿元和8177亿元，分别是2011年的2.28倍和2.8倍。

　　五年来，全市上下大力开发建设兰州新区，在“一张白纸”上描绘蓝图，一个产业新城正在快速崛起。累计完成投资1986.58亿元，基本建成核心区120平方公里的基础设施，形成区内路网482公里，兰秦快速通道、中马铁路建成通车。引进产业项目318个，建成投产49个。综合保税区建成运营，注册企业200多家。建成市体育学院和6所中小学、幼儿园，大型职教园区建设快速推进。完成造林绿化18.3万亩，建成栖霞湖、秦王川国家湿地公园等生态景观。预计今年生产总值达到150亿元，是获批建区时的3.84倍。兰州新区的开发建设，解决了城市发展空间不足的问题，成为疏解老城区功能的主阵地和产业转移的承接地，为城市长远可持续发展开辟了新空间。

　　五年来，全市上下紧紧围绕省会中心城市的发展定位，加快完善综合功能，城市承载力逐步提升。轨道交通1号线一期工程全线开工建设，完成总工程量的76%，2号线一期工程启动建设，全市人民的地铁梦即将变成现实。推进“139”城市骨干路网和“321”环城公路网建设，南山路、北环路2条城区交通大动脉建成通车，南绕城、北绕城东段高速公路加快建设。拓建改造城市主次干道92条，建设黄河大桥5座，建成“上跨下穿”工程4个，新建人行过街天桥地道54座。建成七里河至安宁快速公交专线，开通公共自行车租赁系统和黄河水上公交。积极破解城市安全供水难题，开工建设新的水源地工程。建成餐厨垃圾、生活垃圾、建筑垃圾资源化利用项目，处理水平居于全国前列。实现城区燃煤锅炉天然气全改造。建成黄河风情线健身步道22公里。新建马拉松主题公园等开放景观，城区新增、改造绿地2345.6公顷，绿地率由26.7%提高到32.63%。建成城市规划展览馆，展示了城市历史记忆和建设成就。

　　五年来，全市上下注重统筹城乡发展，努力缩小二元结构差距，农村面貌发生巨大变化。深入推进脱贫攻坚和“双联”行动，制定实施“1＋21”精准脱贫方案，把人力物力财力向贫困地区倾斜，五年整合投入财政资金182亿元，发放精准扶贫贷款13.73亿元。新增高原夏菜、百合、玫瑰、中药材种植面积55.15万亩，累计达到145.21万亩。市级以上农业产业化龙头企业达到133家。新建农村公路2346公里，建制村通畅率达到100%。引洮工程榆中项目开工建设，完成39座大中型泵站改造，农村自来水入户率达到76%，解决了48.6万人饮水安全问题。完成农村危房改造2.3万户。实现贫困村标准化卫生室、文化活动室、村委会、文化广场全覆盖。创建省级美丽乡村35个、市级美丽乡村60个。城乡居民收入比由3.04倍缩小到2.76倍。

　　五年来，全市上下切实保障和改善民生，大力发展各项社会事业，广大群众的获得感明显增加。累计投入资金168亿元，兴办为民实事129件。城乡居民人均可支配收入年均分别增长11.7%和13.87%，预计达到29526元和10679元。新增城镇就业56.09万人。基本建立覆盖城乡的社会保障体系，城乡居民基础养老金提高90.9%，城乡低保补助分别提高85%和188%，城乡医保补助提高90%，新农合参合率达到98.05%。建设保障性住房16.12万套，完成棚户区改造9.2万套。建成城乡公办幼儿园189所、寄宿制学校127所、标准化学校604所，中小学D级危房全面消除，引进北京实验二小等优质教育资源。实施创意文化产业园等文化旅游重点项目。建成市社会福利院老年养护中心。三维数字社会服务管理系统作为国家行业标准在全国推广，兰州入围“第六届中国智慧城市建设50强”。食品安全电子追溯体系建设走在全国前列。安全生产四项控制指标保持平稳和下降。“平安兰州”建设积极推进。依法管理民族宗教事务水平得到提升，全市呈现民族和谐、宗教和顺的良好局面。

　　五年来，全市上下积极推进重点领域改革，不断扩大对外开放，发展活力有效释放。启动兰白科技创新改革试验区建设，建成运营兰州科技大市场，创办兰州科技成果博览会，培育科技企业孵化器27个、新型众创空间66家，挂牌科技成果转化基地38家、研发机构18家，建立7个产业投资基金。“放管服”改革积极推进，取消、调整和下放行政审批事项316项，编制运行“五张清单一张网”，探索了项目建设“模块化”审批模式。“多证合一”等商事制度改革全面实施。建立统一的公共资源交易平台。组建“1＋8”国有资本投资运营公司。积极参与“一带一路”建设，打造丝绸之路经济带核心节点城市。在全国省会城市中率先加入上合组织睦委会。与丝绸之路经济带沿线国家缔结友好城市3个。甘肃(兰州)国际陆港加快建设。开通到中亚、欧洲和南亚的国际货运班列，累计发运115列，南亚班列被国家列为多式联运示范工程。兰州铁路口岸获批开放。启动中韩、中德等国际产业园建设。兰州正在从传统的内陆城市转变为向西开放的前沿高地。

　　五年来，在推动经济社会发展过程中，政府系统坚持不懈加强自身建设，干部作风持续转变。依法接受市人大及其常委会法律监督和工作监督，主动接受市政协民主监督，办理人大代表建议984件、政协委员提案1828件，办复率100%。强化政府立法工作，提请市人大审议地方性法规20部，制定政府规章29件。推进依法行政和政务公开，健全政府法律顾问制度。严格执行中央“八项规定”、国务院“约法三章”和省委“双十条”规定、市委“十四条”规定，深入开展党的群众路线教育实践活动、“三严三实”专题教育和“两学一做”学习教育，解决了一批不严不实的作风问题。坚持“两手抓两手硬、双促进双落实”，从严治党主体责任有效落实，政府系统党风廉政建设得到新的加强。

　　五年来，全市审计、统计、税务、司法、人防、地震、供销、地方志、档案、消防、气象、校外教育和社会科学等工作取得新进展，妇女儿童、残疾人、老龄等事业取得新进步，双拥工作荣获全国双拥模范城“八连冠”。

　　各位代表，五年来兰州各项事业取得的新成就，是省委、省政府和市委坚强领导的结果，是市人大、市政协监督支持和社会各界共同努力的结果，全市上下凝心聚力，克服各种不利因素，为兰州各项事业发展付出了巨大心血和努力。在此，我代表市人民政府，向奋战在全市各条战线的人大代表、政协委员、广大干部群众，向各民主党派、人民团体、离退休老同志，向驻兰部队、武警官兵、公安干警、中央、省属驻兰单位和新闻媒体，以及所有关心、支持和参与兰州建设发展的同志们、朋友们表示衷心的感谢！

　　五年的实践启示我们：加快兰州经济社会发展，必须始终树牢绝对忠诚的政治意识、大局意识、核心意识和看齐意识，坚决有力地贯彻落实中央、省委省政府和市委的各项决策部署，同频共振、步调一致；必须始终扭住发展这个第一要务，充分发挥省会城市的突出优势，调整优化经济结构，加快转变发展方式，提升发展质量效益；必须始终紧盯城市建设中的短板问题，集中力量攻坚克难，完善综合服务功能，提升城市对外形象和影响力；必须始终把握国家向西开放战略机遇，充分发挥兰州独特的区位优势条件，主动融入“一带一路”建设，在向西开放中抢占先机、走向前沿；必须始终坚持以人民为中心的发展思想，统筹城乡发展，全力保障和改善民生，不断增加和提升民生福祉；必须始终强化依法行政，切实加强法治政府建设，提高运用法治思维和法律方式推动工作的能力；必须始终牢记作风建设“永远在路上”，坚持守纪律、讲规矩，着力转作风、强素质，为创造人民满意的发展业绩提供坚强保证。

　　各位代表，过去的成绩值得肯定，但发展中的矛盾和问题更不能忽视。当前，我市经济总量小，发展速度慢，与全国省会城市相比差距较大；产业结构不合理，创新驱动力不强，传统支柱产业层次不高，新的发展动能培育缓慢，经济增长乏力；投资增量和结构性矛盾明显，产业性重大项目少，缺乏体量大、效益高、带动力强的支撑项目；城市基础设施欠账大，城市管理不够严格精细，交通拥堵和环境问题突出；公共服务供给不够均衡，各项民生设施配套总体不足，与广大群众的需求还有差距；一些政府工作人员缺乏强烈的“省会意识”，担当力、执行力、落实力、服务力仍然不够，政务环境还需改善优化。对此，我们要保持清醒头脑，突出问题导向，采取有力措施，认真加以解决。

　　二、今后五年发展的目标任务

　　未来五年的发展，我们既面临严峻挑战，又将迎来难得机遇。虽然宏观经济下行压力依然较大，实现经济平稳较快增长面临不少困难，但随着“五位一体”总体布局和“四个全面”战略布局统筹推进，有利于我们创造和积累加快发展的新优势，后发赶超的时机已经成熟；虽然我市经济发展的结构性矛盾突出，传统动能提升和新动能培育制约因素较多，但随着五大发展理念的深入落实和供给侧结构性改革持续推进，有利于我们适应经济发展新常态，培育新的增长动能，势必推动发展换挡和提质增效；虽然区域发展竞争不断加剧，我市参与区域竞合发展、赢得战略主动挑战很大，但随着“一带一路”战略深入实施，铁路、公路、航空等交通体系日趋完善，有利于我们向东承接产业转移，向西拓展开放空间，进一步形成融合发展的良好局面；虽然我市财力水平与满足人民群众过上美好生活新期待之间的矛盾仍然突出，但随着以人民为中心的发展思想深入人心，有利于我们集中财力办大事、办实事，让城乡群众生活得更加幸福。只要我们积极应对困难挑战，紧紧抓住大好机遇，强化使命担当，凝聚精神力量，自觉奋发作为，就一定能够谱写兰州发展更加灿烂辉煌的美好篇章！

　　前不久召开的市第十三次党代会，描绘了兰州未来五年发展的宏伟蓝图，确立了建设现代化中心城市的战略导向。今后五年的政府工作，要以党的十八大和十八届三中、四中、五中、六中全会精神为指导，深入学习贯彻习近平总书记系列重要讲话精神，坚决落实省委、省政府和市委的部署要求，紧紧围绕“突出‘一条主线’、扭住‘三大任务’、聚焦‘五大发力重点’”的目标任务，立足构建大都市、大产业、大枢纽、大物流、大市场、大平台，着力加快转型发展，着力改善人居环境，着力推进改革创新，着力扩大向西开放，着力增进民生福祉，着力提升行政效能，加快建设经济繁荣、功能齐全、环境优美、文明和谐、富有活力、辐射带动力强的现代化中心城市。

　　今后五年全市经济社会发展的主要目标是：——到2018年，基本实现全面小康社会目标，全市生产总值达到2600亿元以上，城乡居民人均可支配收入分别达到36000元和13000元以上，经济转型实现较大突破，社会和谐度明显提升，城市形象品位进一步彰显，发展的平衡性、协调性、可持续性逐步增强。

　　——到2020年，完成“十三五”规划目标任务，全市生产总值突破3000亿元，城乡居民人均可支配收入分别达到44000元和16000元以上，经济结构更加合理，群众幸福感进一步提升，城市竞争力明显增强。

　　——到2021年，新一届政府届满时，全市生产总值达到3300亿元以上，城乡居民人均可支配收入分别达到48000元和18000元以上，形成一批新兴支柱产业，群众生活更加殷实，基本建成现代化中心城市，兰州在全国发展格局中的地位得到较大提升。

　　围绕上述目标，我们将突出重点，聚焦发力，努力在八个方面实现重大突破：

　　(一)突出创新驱动发展，在提升经济增长内生动力上实现重大突破。坚持把创新作为发展的第一动力，充分发挥省会城市科技资源聚集优势，加快兰白科技创新改革试验区建设，努力建设“创新之城”，使创新创造成为兰州发展的强大引擎。深入推进大众创业、万众创新，着力打造一批低成本、便利化、全要素、开放式的众创空间，催生和壮大新产业、新技术、新业态、新模式。坚持产学研融合发展，强化科技成果转化应用，做大做优战略性新兴产业，加快形成一批新的经济增长点。大力发展文化旅游、商贸物流、电子商务、金融保险、会展经济、健康养生等新兴服务业，把兰州打造成为带动全省、服务西北的现代服务业中心。

　　(二)突出“三区”率先发展，在提升辐射带动能力上实现重大突破。加快推进兰州新区开发建设，构建产城融合新体系，重点打造城市核心区，完善基础设施配套，强化公共服务保障，深化体制机制创新，建设全省改革创新的特区和创新创业的高地。发挥高新区和经济区平台优势，积极开展创新政策先行先试，激发各类创新主体活力，建设开放创新先导区、技术转移集聚区、转型升级引领区，努力实现创新成果倍增、经济总量翻番、综合排名提升，切实增强国家级开发区对全市经济发展的带动能力。

　　(三)突出项目支撑发展，在提升可持续发展后劲上实现重大突破。始终把重大项目建设作为经济社会发展的总抓手，进一步提高项目谋划、争取和落实能力，以大项目拉动大投资、促进大发展，力争五年固定资产投资达到1.2万亿元。推进产业升级项目建设，按照老城区“退二强三”、新区“强二优三”的思路，通过产业延伸和招商引资，实施一批重大产业项目。推进交通枢纽项目建设，实施“6873”交通突破行动和“兰州率先畅通工程”，加快轨道交通建设，实施城市立体交通项目，完善“139”城市骨干路网和“321”环城公路网骨架，构建现代化、立体化、网络化大交通体系。推进民生改善项目建设，在教育、卫生、文化、体育、养老、城市安全等方面实施一批急需的补缺项目，促进基本公共服务均等化。

　　(四)突出攻坚促进发展，在提升城市建设管理水平上实现重大突破。坚持“尊重规律、集约发展、统筹推进”的城市工作总要求，统筹规划、建设、管理三大环节，抓好文明城市、管廊城市、海绵城市、智慧城市建设，加快形成与省会地位相适应的城市功能。全力开展交通拥堵治理，实施路网畅通、公交优先、智慧交管、综合整治工程，力争打通市内所有断头路、瓶颈路、出口路。大力推进棚户区和城中村改造，实施老旧住宅小区综合整治，基本消除棚户区和危旧房。始终把人民群众生命财产安全放在重要位置，有效预防和遏制重特大安全事故发生。深入推进“五城联创”，不断完善城市管理和服务，提高市民文明素质，努力在全社会营造热爱兰州、宣传兰州、维护兰州、建设兰州的浓厚氛围。

　　(五)突出城乡统筹发展，在提升新型城镇化水平上实现重大突破。城镇化是现代化的必由之路，也是推动发展的内需潜力和动能所在。我们将以人的城镇化为核心，加快户籍制度改革，积极推进农业转移人口市民化。加强城镇基础设施建设，提升综合承载能力，把远郊“三县一区”建成功能完善、发展强劲的小城市，把重点小城镇建成各具特色的产业和人口集聚中心。着力增加公共产品和公共服务供给，构建均等化、多层次、宽领域、广覆盖的基本公共服务体系。完善小城镇土地利用机制，创新投融资方式，引导社会资本参与建设，着力破解用地、资金等瓶颈制约，促进小城镇健康持续发展。

　　(六)突出文化推动发展，在提升城市魅力形象上实现重大突破。独具特色、悠久深厚的黄河文化、丝路文化、民族文化，是大自然和历史赐予兰州的宝贵财富。我们将着力加强文化软实力建设，激活城市文化基因，涵养城市精神力量，延续城市历史文脉，绽放兰州文化的绚丽色彩。把文化旅游作为经济社会发展的重要“助推器”，挖掘城市文化内涵，规划建设一批大景区和特色景点，打造文化旅游标志性工程，全面提高文化旅游展示力、感染力和吸引力。促进兰州都市文化产业区建设，培育壮大文化创意、节庆会展、演艺娱乐、出版发行等文化产业集群。通过文化的大发展，让城市散发文化的温度，让文化成为城市的灵魂。

　　(七)突出绿色承载发展，在提升山水城市品质上实现重大突破。生态脆弱、环境承载压力大，是兰州发展的重大制约瓶颈。我们将倍加呵护兰州的山水资源，加大生态建设和环境保护力度，加快构建国家生态文明先行示范区，让兰州的天更蓝、地更绿、水更清，努力建设西部特色山水城市。推进大气污染防治标准化、精细化、常态化，实现空气质量在全国重点城市排名中稳步上升，让全市人民呼吸上更加清洁的空气。推进河洪道综合整治和景观提升，将市区河洪道打造成市民的休闲娱乐区、城市景观的展示区。推进绿色循环低碳发展，建设一批循环产业链示范工程，提高垃圾分类处置和资源化利用水平，全力打造低碳城市。

　　(八)突出开放引领发展，在提升外向型经济水平上实现重大突破。立足区位优势，推进互联互通，着力打造丝绸之路经济带核心节点城市。建成甘肃(兰州)国际陆港，发挥综合保税区等国家级政策平台功能，深化与俄罗斯、中西亚及欧洲的商业贸易，推动国际产能合作，争取设立兰州自由贸易区。推进文化旅游西进工程，构建丝绸之路经济带人文交流大平台。力争在国家向西开放战略格局中，兰州成为“一带一路”的重要平台和前沿高地。

　　各位代表，面对未来五年的美好蓝图，我们激情澎湃、信心满怀。只要我们紧紧围绕市第十三次党代会确定的目标任务，凝心聚力，砥砺奋进，就一定能再铸兰州发展的新辉煌，就一定能让全市人民生活得更加幸福美好！

　　三、2017年的重点工作

　　各位代表！2017年是新一届政府的起步之年。我们将牢牢扭住建设现代化中心城市的目标，坚定信心，增强定力，顺势而为，精准施策，努力推动各项工作迈上新台阶。经济社会发展的主要预期目标是：生产总值增长8%以上；第一产业增加值增长5.2%；第二产业增加值增长7%，其中规模以上工业增加值增长6%；第三产业增加值增长9.5%；固定资产投资增长10%以上；社会消费品零售总额增长9%；一般公共预算收入增长10%；城镇居民和农村居民人均可支配收入分别增长9.5%和10.5%；城镇登记失业率控制在4%以内；居民消费价格涨幅控制在3%以内；各项约束性指标完成国家和省上下达的目标任务。

　　实现上述目标，做好明年政府工作，最关键的是稳中求进、转型突破，改革创新、破解难题。稳中求进，就是牢固树立和贯彻落实新发展理念，科学把握增长速度和质量的平衡点、经济发展和民生改善的契合点，力争各项预期目标增速在合理区间运行；转型突破，就是把经济转型升级摆在更加突出的位置，既努力稳住传统产业存量，更注重培植新兴产业增量，加快塑造产业核心竞争力；改革创新，就是按照中央、省上和市委关于全面深化改革的要求，把改革创新贯穿到政府工作全过程，坚定不移抓好重点领域改革，不断释放改革红利，为经济社会发展增添活力；破解难题，就是强化补短板的思维，紧盯经济社会发展的薄弱环节，统筹加强公共设施、公共服务、公共管理、公共安全，尤其是针对交通拥堵、环境脏乱差、违法建设、各种线缆乱架乱布等突出“城市病”，集中开展强有力的整治活动。

　　在明年的工作中，我们将重点突出产业升级“七大工程”、城市环境“三大整治攻坚战”、城市形象“六个改造提升”、对外开放“两大平台建设”、为民兴办“25件实事”，以点带面、推动全局。

　　(一)围绕经济转型升级，聚焦发力“七大工程”。主动适应经济发展新常态，以推进供给侧结构性改革为主线，大力振兴实体经济，实施产业升级“七大工程”，加快培育新产业、新业态、新模式，强化经济发展新动能。

　　实施创新驱动工程。提升兰白科技创新改革试验区集聚、示范、辐射和带动功能，争取获批国家自主创新示范区。着力打造兰州新区产业孵化中心，构建“创业苗圃＋孵化器＋加速器＋产业园”的全产业链孵化链条。培育发展各类众创孵化平台，为创业者提供优质服务，打造充满活力的创新创业生态圈。继续推进城关省级双创示范区建设。深化院地校企合作，促进新产品研发和产业化发展。办好2017兰州科技成果博览会，促进产业、技术、资本深度融合。全力支持科技型中小企业发展，扶持培育10家以上拥有自主知识产权、核心竞争力强的优势企业。

　　实施“四千七百”产业链延伸壮大工程。坚持传统产业改造与新兴产业培育相结合，着力打造石油化工、新材料、装备制造、电子信息(大数据)四个千亿级和有色冶金、建材、烟草、生物医药、新能源、节能环保、轻工及食品七个百亿级产业链，优化工业结构和产业体系。石油化工产业重点配合实施兰州石化技术改造工程，争取原油加工产能稳定在1000万吨；新材料产业重点做大科天化工水性材料、方大炭素核石墨等特色产业链；装备制造产业重点抓好节能与新能源汽车、正威精铜杆等项目建设；电子信息(大数据)产业重点以“三维数字”技术为基础，加快建设大数据交易中心、大数据产业园、电子商务孵化园等项目，打造国家大数据综合试验区。有色冶金产业重点推进钢铁、铝精深加工产业链延伸，实施榆钢钢结构、兰亚铝业等项目；建材产业重点抓好西部建材商品混凝土、京兰环保新型干法水泥等项目建设；烟草产业重点推进甘肃烟草卷烟生产线技术改造项目；生物医药产业重点支持佛慈制药、陇神戎发等企业发展现代创新中药，支持中成药大品种二次开发；新能源产业重点加快金川科技园二次电池材料示范园等项目建设；节能环保产业重点结合“城市矿产”示范基地创建工作，推进红古再生资源产业园、华壹静脉产业园等项目建设；轻工及食品产业重点发展纺织服装业，提升百合、玫瑰等特色农产品深加工水平。

　　实施现代服务业优化提升工程。坚持“退二强三”，促进主城区服务业提档升级。抓好西客站、马滩、安宁等新兴高端商务区建设。加快推进毅德、北龙口、省物产集团、五矿钢铁等物流集散中心建设。促进商贸市场转型升级，加快城区各类批发市场“出城入园”。大力发展金融服务业，力争兰州银行在A股成功上市，扶持一批中小企业在新三板挂牌。抓好丝路电商产业园建设，支持百合生活网等本土电商发展，力争电子商务交易额增长35%。大力发展会展经济，举办重点展会65个，实现交易额90亿元以上。

　　实施文化旅游突围突破工程。坚持以文化为灵魂、以旅游为载体、以项目为支撑，打响“黄河之都、金城兰州”的城市形象品牌，把文化旅游发展成为转型升级的重要支柱产业。加快黄河风情线和兴隆山两个大景区，以及大兰山、吐鲁沟、丹霞地质公园等特色景区建设。推进兰州老街、黄河夜景开发、陇香源民俗文化小镇、游客集散服务中心、仁寿山和天斧沙宫等重点项目建设。做大做强兰州创意文化产业园、飞天文化产业园、华夏收藏文化博览园等现代文化产业园区。实施黄河兰州段水域开发提升工程，建设集陕甘青蒙民俗文化于一体的黄河水上展演区。办好第七届中国(兰州)黄河文化旅游节、第二届黄河母亲节等文化旅游品牌节会。大力发展全域旅游，促进旅游与相关行业融合发展。力争文化产业增加值增长18%，接待国内外游客增长22%，旅游收入增长24%。

　　实施县域经济振兴工程。制定县域经济振兴发展规划，支持县区走出各具特色、各有优势的发展路子，让县域经济成为充满生机的经济体。把园区作为壮大县域经济的重要抓手，明晰发展定位、功能布局和主导产业，做大做强园区经济。调整优化农业产业结构，发展壮大七里河百合、永登玫瑰、榆中高原夏菜、皋兰瓜果、红古设施农业等特色产业，增加绿色优质农产品供给，加大品牌宣传和推广力度，提升规模化、基地化、品牌化、市场化水平。加大政策扶持力度，培育一批带动作用大、核心竞争力强的农业产业化龙头企业，促进现代都市农业加快发展。

　　实施非公经济多元发展工程。鼓励非公经济放心、放手、放胆发展，继续实施创业带动就业“万企计划”，重点扶持发展一批潜力大、成长性好的初创微型企业。健全重大项目公开引入社会投资机制，支持非公企业通过PPP、收购股权、参股、控股、租赁和购买服务等形式进入公共行业领域，拓宽民营资本投资渠道。注重发展混合所有制经济，引导民营企业参与市属国有企业改革。

　　实施全民绿化工程。保护生态环境就是保护生产力，改善生态环境就是发展生产力。我们将牢固树立“绿水青山就是金山银山”的理念，坚持政府引导与社会参与相结合，动员机关、部队、学校、农村和社会各方面广泛参与，营造人人参与、人人尽力、人人享有的造林绿化氛围，大力发展兰州的绿色空间。力争今后五年新增造林绿化60万亩，每年实施12万亩。重点推进黄河两岸、南北两山、农村宜林荒山造林绿化，打造城乡绿色屏障。结合林权制度改革，使造林绿化与产业富民有机融合，开展试点示范，发展林业经济，促进群众增收。

　　(二)发挥国家平台优势，培育壮大“三区”经济增长极。坚持以产促城、产城融合，掀起兰州新区、高新区和经济区新一轮开发建设热潮，做大产业、完善配套、优化环境、提升人气，彰显经济建设主战场地位，当好全市率先发展“排头兵”。

　　推动兰州新区产城融合发展。坚持“新”字当头，运用新机制，探索新模式，发展新业态，壮大新产业，力求新突破。继续完善基础设施功能，抓好景中高速、中川至朱家窑铁路建设，加快构建骨架路网体系，实施城市功能配套重点项目。坚持“强二优三”，把产业集聚作为新区开发的生命线，延伸拓展“6＋4”产业链，引进产业项目30个以上，建成投产20个以上。加快长城影视城、西部国际文商旅产业园、环球国际嘉年华、中俄文化交流中心等大型文化旅游园区建设。完善公共服务，加快职教园区建设，建成一批具有较高水平的知名学校。坚持节约集约利用土地，严控增量、盘活存量、优化结构，提高项目建设投资强度。研究出台购房、就业、落户等倾斜性政策，引导人口向新区转移聚集，打造人才、产业、经济新高地。

　　激发高新区和经济区发展活力。高新区坚持以“高”为主轴、以“新”为目标，抓好一批科技企业孵化器建设，建好“创新大街”和“创新工场”，加快榆中园区配套公共服务设施建设，吸引科研院所高精尖技术向东部科技城集聚，打造产城融合发展示范区。经济区着力推进“一区五园”协调发展，壮大园区主导产业规模，加快西部药谷等重点项目建设，扶优做强佛慈制药等16户骨干企业，提升产业核心竞争力。

　　持续强化招商引资工作。开展“招商引资促进年”活动，编制招商引资导则，增强招商引资精准性和实效性。强化联动招商，在“一带一路”节点城市推介兰州，互通产业新模式，互联交通新路径。强化产业招商，加大与行业领军企业对接力度，着力引进一批科技含量高、投资体量大、带动能力强的优质项目。强化环境招商，营造“投资放心、发展舒心、收获开心”的投资氛围，创造一流的政务、法制、市场和社会环境，以最优质的服务吸引客商、留住客商。强化节会招商，办好第23届兰洽会，深度参与民企陇上行、浙商陇上行、陇商回家乡等系列活动，力争项目签约和引进资金再创新高。

　　(三)顺应城市发展规律，做优做美做靓城市。坚持最严的规划、最严的执法、最严的土地管理，高水平规划城市，高质量建设城市，高标准管理城市，努力创造更加便捷、更加舒心、更加美好的人居环境。

　　全力巩固提升“兰州蓝”。推动大气污染治理向常态化转变，构建完备、标准、规范的治理体系。完成兰州石化重催脱硝、炼油污水处理、废气净化、催化厂工艺尾气治理和西固电厂超低排放等重点深度治理项目，力争国电兰州热电“上大压小”异地扩建项目建成投产。细化扬尘管控，在重点工地安装扬尘智能监控设备，实时监测扬尘数据。加强城区渣土运输统一管理，启动运营渣土车等移动源排污权交易试点工作。淘汰市内所有黄标车，全市油车同步施行国五标准准入。抓好“净水”、“净土”工程，继续开展黄河枯水期跨流域水污染联防联控，推进兰州石化地下水修复等重点项目，启动土壤环境保护和综合治理工作。

　　打好城市环境“三大整治攻坚战”。一是打好交通拥堵大整治攻坚战。强化顶层设计，坚持建管并举，打通一批断头路，实施微循环和立体化改造，推进公交优先，加强智能交通、静态交通和慢行系统建设，全面改进和加强交通执法，推进交通文明进社区、进学校、进机关、进部队，构建全民动员、全员参与、共管共治、共建共享的交通治理新格局，力争城区交通拥堵明显改善。二是打好城乡环境大整治攻坚战。针对城市“七乱”和农村“十乱”问题，坚持城乡同步、点面结合、标本兼治，紧扣整治重点，做到多措并举，持续聚焦发力，推动城乡环境面貌明显改观。三是打好背街小巷大整治攻坚战。立足解决困扰群众的“家门口”问题，铺平群众脚下路，点亮群众门前灯，美化群众居住地，改善背街小巷人居环境和出行条件，大力提高群众生活舒适度。

　　加快补齐城市功能短板。建成城市轨道交通1号线一期工程，加快建设2号线一期工程，开工建设4号线工程，开展3号线前期工作。启动雁青、古浪路黄河大桥建设和东岗立交桥改造。开展南山路高架快速通道研究论证工作，构建城市快速路网。新建一批大型立体停车场。改造完善城市供水管网。实施雨污管网分流改造。整治城市黑臭水体。扩建七里河安宁、盐场2个污水处理厂。推进马滩片区6条道路地下综合管廊建设工程，申报国家综合管廊试点城市，探索开展海绵城市建设。

　　推进城市形象“六个改造提升”。一是推进城市风貌改造提升。编制城市风貌规划，提高城市设计水平，确定富有兰州特色的城市建筑风格和色彩色调，在规划执行中严格落实。二是推进黄河风情线改造提升。坚持整体规划、分步推进，突出风格特色，彰显黄河文化，实施夜景亮化，打造璀璨亮丽、独具魅力的滨河景观长廊。三是推进园林绿化改造提升。启动彭家坪中央生态公园和金城公园二期项目，建设改造一批小游园、小广场、小绿地，鼓励推广庭院绿化、立体绿化、屋顶绿化、见缝插绿、拆墙透绿，新增、改造绿地80公顷。四是推进东方红广场改造提升。精心规划设计，全面升级铺装、绿化、灯光、建筑立面等设施，提升综合功能和景观品质，做美做优“城市客厅”。五是推进城市出入口改造提升。突出美化、亮化、绿化、净化，实施山体绿化、治荒治斑、园林造景、水面水系、文化提升和环境整治，打造有特色、多景点、上层次的城市“第一印象”。六是推进坪台地区改造提升。结合地质灾害防治，统筹谋划坪台地区开发改造和环境提升，先行启动伏龙坪地质灾害综合整治。

　　释放小城镇发展活力。小城镇一头连着城市，一头连着农村，是撬动新型城镇化的支点。我们要着力打造小城镇经济，培育发展休闲旅游、传统文化、农副产品加工、科教服务、工业服务等特色产业，探索推进“智慧小镇”建设，让小城镇成为推动经济发展新的闪亮点和增长点。坚持规划引领、以“特”兴镇，加大政策支持力度，完善基础设施功能，健全公共服务体系，提升小城镇承载力、集聚力、带动力。支持青城古镇国家级特色小镇建设，争取河口黄河风情小镇、什川梨园小镇入选国家特色小镇，抓好阿干、连城、红城、苦水、金崖等特色小城镇建设。

　　(四)加快改革开放步伐，打造向西开放战略平台。始终把改革开放作为发展进步的“关键一招”，更好发挥改革的牵引作用和开放的引领作用，持续深入推进各项改革，深度融入“一带一路”建设，努力做到改革增动力、开放添活力。

　　抓好重点领域改革。落实供给侧结构性改革“三去一降一补”任务，主动减量、优化存量、引导增量。继续深化“放管服”改革，推进“三减一压缩”，着力提升审批效率。完善国有资产管理体制，以管资本为主加强国有资产监管。分类推进市属国有企业改革。深化投融资体制改革，大力推广PPP融资模式。完善政府债务限额管理、预算管理、风险预警和监督考核制度。健全生态补偿机制，推广碳排放权、排污权、水权等交易试点。

　　建好两大开放平台。完善甘肃(兰州)国际陆港交通设施，建成西行线、五〇四立交桥、北滨河路西延段、国道309线西固段和9条内部道路，加快推进铁路集装箱货运中心、铁路口岸监管场所、保税物流中心(B型)、陆港物流信息中心、多式联运物流园建设。提升新区综合保税区运营水平，加快跨境金融结算中心、跨境电商平台建设，设立汽车整车、鲜活肉禽等特殊功能口岸，促进综合保税区与中川航空港、铁路口岸联动发展。

　　培育贸易畅通新优势。推动兰州至中亚、欧洲、南亚国际货运班列及兰州至迪拜国际货运包机常态化运营，争取获批建设中欧国际货运班列编组枢纽和物流集散转运中心。拓展境外营销网络建设，支持知豆电动汽车开拓国外市场，推动小型客车、特种车辆出口。实施“互联网＋外贸”行动，加快推进跨境电子商务和综合服务平台建设，扶持培育一批跨境电商企业。建设进口商品展示中心，打造国际商品集散地。

　　拓展深化国际产能合作。加快建设中韩、中德、中哈、中以等特色产业园，引进一批电子产品、精密机械等项目，打造国际中小企业合作示范基地。支持兰石集团、大成科技等企业建设面向中西亚、中东欧市场的出口基地。鼓励支持有条件的企业在境外投资建厂，通过境外上市、跨区域兼并重组等方式参与国际竞争。

　　推进人文交流合作。巩固发展友好城市关系，深化拓展交流合作领域，新缔结2—3个友好交流城市。在丝绸之路沿线国家开展文化交流活动，组织《大梦敦煌》等文化精品赴外演出，推进和扩大文化贸易。拓展国际文化旅游市场，在白俄罗斯、塔吉克斯坦、捷克等国家设立兰州文化旅游推广联络处。同丝绸之路沿线国家城市开展教学研究、人才培养、师生互换、教育展览等交流合作，促进双边学生留学和学者互访。

　　(五)顺应人民群众期盼，进一步保障和改善民生。始终把增进人民福祉作为工作的出发点和落脚点，把群众的需求和满意作为工作的标准和要求，持续加大保障和改善民生的力度，努力让人民群众有更多的获得感和幸福感。

　　立足民生之急，深化精准扶贫脱贫。推进220个小康村建设，做到生态宜居环境美、兴业富民生活美、文明和谐乡风美、干净整洁村庄美。大力发展现代农业，实施都市农业、高原夏菜、百合、玫瑰、中药材五大发展规划，抓好10个省级现代农业示范园区建设，新增高原夏菜、百合、玫瑰、中药材种植面积7万亩。新发展市级以上农业产业化龙头企业15家、示范性农民合作社50家、家庭农场30家。推广“互联网＋农业”，村级电子商务服务点覆盖率达到80%。建成14个省级美丽乡村、20个市级美丽乡村、60个环境整洁村。

　　立足民生之本，加强就业和社会保障。继续实施“泛海扬帆·兰州启航”大学生资助扶持计划，促进高校毕业生就业。支持下乡返乡人员创业创新。抓好退伍军人、残疾人、困难家庭等重点人群就业。培育2至3个“万人”就地转移劳务基地。完善被征地农民养老保险制度。建成异地就医结算平台网络系统，建立统一的城乡医保制度，推进医疗、医保、医药“三医联动”，全面实行分级诊疗制度。全力推进棚户区改造和城中村改造，不断改善困难群众住房条件。

　　立足民生之基，推进社会事业繁荣发展。始终把教育摆在优先发展的战略地位，合理规划学校布局，促进教育均衡发展，扩大一体化办学范围，加快建设兰州二中雁滩分校、兰州五十一中九州分校等项目。出台《健康兰州2030规划》，推动市中医院、市妇幼保健院异地新建。建设医养结合服务机构4家。启动市级“五馆合一”项目，实施县区“三馆”改造提升。开展“百千万”文化惠民工程。继续办好兰州国际马拉松赛。启动奥体中心“一场一馆”建设。全面推广普及三维市民卡。驰而不息抓好全国文明城市创建工作，不断提升城市文明水平。做好“双拥”工作，巩固发展军政军民团结。深入开展“七五”普法。积极推进防范和遏制重特大安全生产事故试点工作。创建国家食品安全城市。严厉打击违法犯罪活动，创造和谐稳定的社会环境。

　　立足民生之要，扎实办好为民实事。社会保障方面，提高城市低保标准10%；提高农村低保标准20.56%；农村五保集中供养补助标准由年人均5900元提高到6650元，分散供养补助标准由年人均4825元提高到5414元；为1379户计划生育“失独”家庭办理综合保险。社会事业方面，建成50所中小学标准化校园足球场；建成40个城乡社区老年人日间照料中心；建成200个全民健身场地；改造提升五泉山、白塔山公园。农村惠民方面，新建农村公路300公里，重点养护600公里；实施1.46万农村人口饮水安全工程。城市惠民方面，启动建设雁滩中心公园；整治提升927条背街小巷；新建10座过街人行天桥；新建公共停车泊位1.7万个；建成水源地项目。安居保障方面，实施3.05万户棚户区改造；整治改造40个“三不管”楼院；新建和改造主城区老旧供热管网80公里。“双创”方面，向小微企业提供信用贷款2.4亿元；城镇新增就业9万人，安置困难群体就业5000人，培训各类劳动力3.4万人。社会管理方面，建成公共安全视频监控联网应用项目一期工程。便民服务方面，新建和改造提升8个便民市场；创建50家“放心粮店”；改造建设146个村级供销综合服务社；建成国际高原夏菜副食品采购中心一期工程。

　　各位代表，做好明年工作事关“十三五”发展大局，事关全面建成小康社会进程，我们将紧盯目标、创新实干，踏石留印、抓铁有痕，向全市人民交出一份满意的答卷！

　　四、加强政府自身建设

　　全面实现市第十三次党代会确定的目标任务，新一届政府使命光荣、责任重大。我们将始终牢记为民、务实、清廉的宗旨，践行忠诚、干净、担当的要求，不断提高驾驭经济、管理社会、推进改革、服务群众的能力，建设人民满意的政府。

　　(一)我们要以坚定的信念忠诚担当。始终树牢政治意识、大局意识、核心意识、看齐意识，把对党忠诚、对国家忠诚、对人民忠诚、对事业忠诚体现到实际工作中，认真履职尽责，提升工作境界。更加强化责任担当，切实担负起推进改革发展的重任，面对矛盾问题不回避、不掩饰、不懈怠，善于调动各方面力量攻坚克难。努力以更严的要求、更高的标准推动工作，在抓落实、促发展上争一流、见实效，为全省经济社会发展出大力、作贡献。

　　(二)我们要以崇高的追求创新谋事。坚持“依靠学习走向未来”，培养战略思维和世界眼光，准确研判把握发展形势，以更高的层次、更宽的视野审视发展、谋划工作。把解放思想作为谋事干事的强大武器，冲破思想观念的束缚，脱离平庸无为的状态，打开思路谋划和推动发展，精准对接上级的部署要求，借助各种有利的机遇条件，加快发展步伐，提升发展层次。把五大发展理念贯穿到发展的全过程，在体制机制、工作思路、措施办法等方面勇于创新，努力走出新路、干出实绩、创出亮点。

　　(三)我们要以高度的自觉服务大局。省会城市肩负着引领发展、服务全局的神圣职责，全体政府工作人员必须树立强烈的“省会意识”，把兰州的发展置于全省发展大局之中，增强中心带动的责任感，催生中心带动的凝聚力，激发中心带动的创造力，培植中心带动的竞争力。牢固树立“大服务”的理念，主动为省直部门单位、中央在兰企事业单位、部队、投资客商以及全社会搞好服务，以服务推动发展、以服务引导投资、以服务促进建设。坚持全心全意为人民服务的根本宗旨，把群众的评价作为衡量工作的重要标尺，真心实意为群众办实事、做好事、解难事，做群众的“贴心人”，当群众的“服务员”。明年，市政府将设立“兰州市民网”，充分听取广大群众对政府工作的意见建议，认真做好办理回复工作，做到件件有着落、事事有回音，使政民互动机制更加畅通完善，让政府工作更加顺民意、暖民心。

　　(四)我们要以严格的规范依法行政。坚决服从市委领导，自觉接受市人大法律监督、工作监督和市政协民主监督，虚心接受各民主党派监督，落实重大事项向市人大报告清单制度，提高人大代表议案、建议和政协委员提案办理质量。坚持把法治精神、法治思维、法治方式融入政府工作全过程，健全完善公众参与、专家论证、风险评估、合法性审查、集体讨论决定的重大决策机制，做到法定职责必须为、法无授权不可为。强化政务公开、政事公开，坚持以公开为原则、不公开为例外，及时晒出政府重大事项和重点工作，保障群众的知情权、参与权、表达权和监督权，让政府更好地服务人民，让人民更好地监督政府。

　　(五)我们要以勤勉的精神一心实干。牢记“空谈误国、实干兴邦”，坚持勤奋理事、激情干事、努力成事，大力倡导说了就办、定了就干、干就干好的实干精神，不务虚功、不干虚事、不慕虚名，对确定的重点工作和重大事项以“钉钉子”精神抓好落实，坚决不打折扣。践行“一线工作法”，把工作的重心下沉到基层，多在一线掌握情况，多在一线解决问题，多在一线推动工作。强化行政问效，严肃查处不作为、慢作为、乱作为等行为，杜绝慵懒散慢现象，努力以政府的勤奋指数，提升群众的幸福指数和城市的繁荣指数。

　　(六)我们要以刚性的约束廉洁从政。始终把纪律挺在前面，把规矩记在心上，把全部心思用在干事创业上，把满腔热情倾注在为民造福上，严格遵守党的各项纪律规定，组织上讲服从、行动上讲纪律，做到遵守纪律没有特权、执行纪律没有例外。严格落实全面从严治党主体责任，切实加强政府系统党风廉政建设，巩固深化作风建设成果，抓好政府工作人员教育管理，多打“预防针”，筑牢“防火墙”，加大对项目建设、土地管理、公共资源交易等重点领域的监管力度，防止发生违纪违规行为。强化行政监察和审计监督，深入开展专项治理，坚决纠正不正之风，确保干部清正、政府清廉、政治清明。

　　各位代表，建设经济繁荣、功能齐全、环境优美、文明和谐、富有活力、辐射带动力强的现代化中心城市的蓝图已经绘就，让我们在省委、省政府和市委的坚强领导下，真情对待我们的城市，痴情对待我们的事业，激情对待我们的工作，深情对待我们的群众，奋力开创兰州全面建成小康社会新局面，不断谱写全市人民美好生活的新篇章！
